# A Comparative Study to Assess the Prevalence, Knowledge of Impact, and Practice of Self-Medication Among Adults in Urban and Rural Communities in Bangalore

**DOI:** 10.7759/cureus.39672

**Published:** 2023-05-29

**Authors:** Prasanna Samuvel Babu, Venkatesan Balu, Bhima Uma Maheswari, Channabasappa K M, Pankaja K E, Embhah Dkhar

**Affiliations:** 1 Medical Surgical Nursing, Padmashree Institute of Nursing, Rajiv Gandhi University of Health Sciences, Bangalore, IND; 2 Child Health Nursing, Padmashree Institute of Nursing, Rajiv Gandhi University of Health Sciences, Bangalore, IND; 3 Mental Health Nursing, Padmashree Institute of Nursing, Rajiv Gandhi University of Health Sciences, Bangalore, IND

**Keywords:** knowledge of impact, practice and adults, adverse effects of medication, consumption of medication, prevalence, self-medication

## Abstract

Background: Self-medication is the act of consuming medicines at the individual's own suggestion or recommendation by a family member, a friend, or untrained or unqualified health care personnel. Practice of self-medication differs among individuals and is influenced by several factors like age, educational status, gender, family monthly income, level of knowledge, and non-chronic illness.

Aim: This study aims to compare the prevalence, knowledge of impact, and practice of self-medication among adults in urban and rural communities.

Materials and methods: A non-experimental comparative study was conducted among adults in urban and rural communities practicing self-medication. In this study, the target population is aged between 21 and 60 years. The sample size is 50 urban adults and 50 rural adults. A convenient sampling technique method was used. The prevalence was assessed through a survey questionnaire. The self-structured questionnaire was used to assess the knowledge of impact, and a non-observational checklist was used to assess the practice which was adopted by the research investigator.

Results: The present study results showed that the majority had inadequate knowledge (88%) regarding consuming self-medication in rural adults, there was overuse of self-medication practice (64%) in rural adults, and there was moderate usage of self-medication practice (64%) in urban adults. There was a statistically significant difference between knowledge and practice regarding self-medication among adults in urban and rural communities which was highly significant at p<0.05.

Conclusion: In the present study, comparison of the results of knowledge of impact and practice of self-medication among urban and rural adults revealed that urban adults have better adequate knowledge of impact of self-medication which helps them to practice moderate usage of self-medication.

## Introduction

Self-medication is the act of consuming medicines at the individual's own suggestion or recommendation by a family member, a friend, an untrained person, or unqualified health care personnel [1]. In the present situation, self-medication has become the most important part of the health care delivery system. Current scenarios in developing countries also show that most individuals consume self-medication around 60-80% of the time to treat illness [2]. According to data from India, the rise in self-medication is a result of family members' illnesses rising health care costs and limited access to health care facilities, particularly in rural areas. Individuals with any illness who are in the early stages of it choose to take self-medication to get better [3]. Lack of family support, lack of time, distances between medical facilities, financial constraints, prolonged wait times for doctors, prior illnesses treated with self-medication, and rising costs of medical professionals are among the causes of self-medication [3].

Self-care helps individuals take responsibility for and have self-confidence in their own health. Individuals perceive that consuming self-medication improves their self-care; it has merits and demerits [4]. The prevalence of self-medication practice among medical students in different nations was observed. Students take medication for pain, fever, respiratory infections, and headaches. The most common drugs used were painkillers, antipyretics, and antibiotics [5]. As per data, the prevalence of self-medication in various countries shows African countries (81%), Europe (68%), Nepal (59%), Sudan (73%), Cameroun (55%), Kuwait (92%), and India (31%), respectively [6].

In our routine lives, we practice self-medication to treat minor illnesses. Self-medication is an individual's decision to treat common minor symptoms. Worldwide self-medication causes human resistance to antibiotics [7]. Consuming self-medication differs among individuals and is influenced by several factors like age, educational status, gender, family monthly income, level of knowledge, and non-chronic illness [8]. Painkillers and antibiotics are commonly used for self-medication [9].

Adverse reactions to self-medications cause damage to the heart, GI system, kidneys, liver, and other organs [10]. The elderly population experienced age-related physical and physiological changes and age-related illness which made them too vulnerable to take self-medication to treat their illness [11]. According to the WHO report, individuals who consume self-medication have a lack of knowledge which has the impact of various possible risks, including misuse of drugs, over dosages, improper self-diagnosis, long duration of drug usage, lack of caution, and polypharmacy [12].

It has been observed that consuming self-medication has become a trend with an increased level of practice in developed and developing countries. Appropriate practice of self-medication helps in the prevention and management of acute illness without physician consultation [13]. Generally, self-medication takes care of minor ailments [14]. Research studies advised that consuming antibiotics without consulting a physician frequently triggers antibiotic resistance and they had poor knowledge about antibiotic practices like dose and course which was observed during the COVID-19 situation [15]. Adults practice self-medication based on their previous experiences and advice from family or friends, without consulting a physician to directly purchase medicine from a pharmacy [16,17].

Self-medication has both benefits and adverse reactions on individuals. The main negative impacts of self-medication are many, but few need more concern in terms of health. Health care professionals need to conduct awareness programs to make aware to the public of this topic to prevent self-medication-induced negative impacts. The public who consumed self-medication had inadequate knowledge of negative impact. As per data worldwide and in developing countries, the majority of people consume self-medication which increased in the past decades; in the Indian context 31% of people consume self-medication and the impact of self-medication is highly dangerous to public health [10,11]. In India, very few studies were focused on this topic. Hence, research investigators felt there is a need in this area to assess the level of knowledge of impact and practice of self-medication among adults in urban and rural communities [6]. 

## Materials and methods

Research approach and design

Quantitative research was adopted to assess the prevalence, knowledge of impact, and practice of self-medication among adults in urban and rural communities. Non-experimental-descriptive comparative study design was adopted in this study. A survey questionnaire was used for prevalence, a self-structured questionnaire was prepared for assessing the level of knowledge of impact, and a non-observational checklist was prepared for assessing the level of practice of self-medication. Research tools were prepared by the research investigator. The entire duration of data collection was one month.

Ethical consideration

The present study was accepted by the ethical committee of 'Padmashree Institute of Nursing' and official approval was taken from the concerned authority of Upanagara PHC and Sulikere PHC on 02-02-2021 with registration number 05_N316_103829, and consent was obtained from the study samples.

Pilot study****

The pilot study was conducted in the urban Kengeri community and rural Byurahalli community with a duration of one week, and the study was found feasible to conduct the main study. 

Setting and sampling

**Figure 1:**
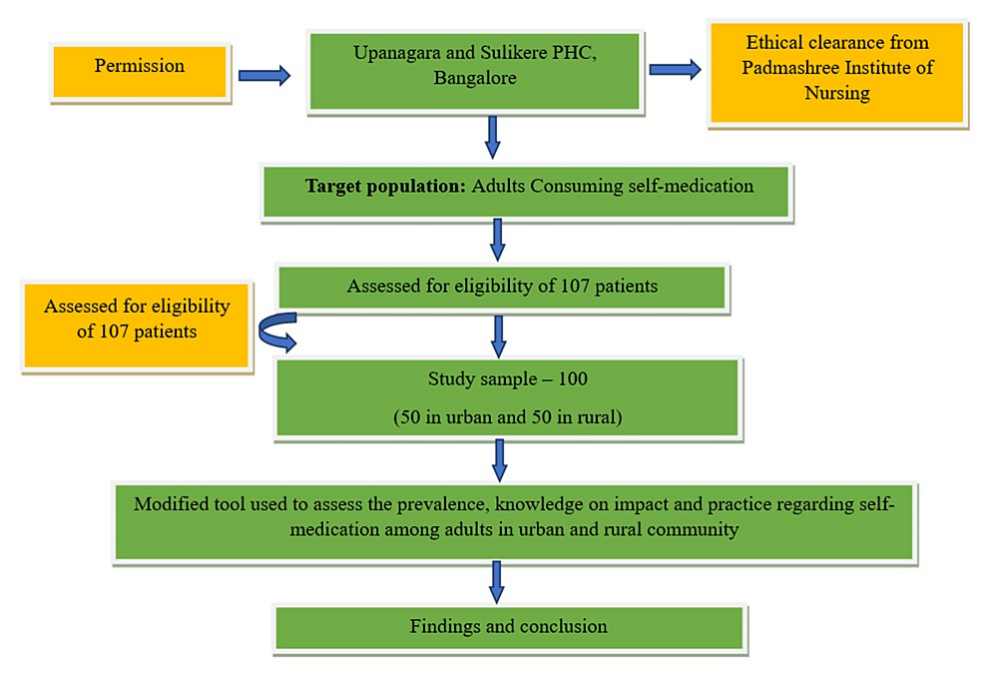
Data collection

The present study was conducted in the urban Upanagara community and rural Sulikere community, Bangalore, from 15th March 2021 to 18th April 2021. The target population was adults who consume self-medication in urban and rural communities. An eligible total number of 108 adults who consume self-medication were chosen, and out of which, 100 were selected (50 urban adults and 50 rural adults) for the study using a non-probability convenient sampling technique (Figure 1).

Sample size estimation

The sample size of the study was calculated considering knowledge as a primary outcome variable. A similar study was conducted by James et al. [18]. The sample size was estimated using power analysis (α=5% and power(1-β)=80%) and the effect size was 0.68. A total of 90 subjects were needed to achieve a significance of 0.05. The study required 90 subjects. Ten percent was added for attrition. Thus, the total samples required are 100 (urban 50 + rural 50).

Measurement outcomes

Development and description of the tools consist of four sections. Section-A was composed of seven items seeking information on demographic data like age, gender, religion, educational status, occupation, monthly income, and marital status. Section-B was a survey questionnaire used to assess the prevalence of the tool which consists of seven items. Section-C, a self-structured questionnaire, was used to assess the level of knowledge of impact of self-medication. The tool consists of 20 MCQs. Each right answer carries one mark and the wrong answer score carries 0 and scoring interpretation of the tool obtained is categorized as follows: inadequate knowledge of the impact score (<10), moderate knowledge of the impact score (10-15), and adequate knowledge of the impact score (>15). The reliability of the tool was obtained using the split-half method, and the score obtained was 0.76 which indicates that the tool is reliable. Section-D was a non-observational checklist used to assess the level of practice of self-medication. The tool consists of 14 statements on often score =3, sometimes =2, and never = 1. Statements are mixed aspects of positive and negative. The maximum score of the tool was 42. Score interpretation obtained was categorized as follows: less usage of self-medication practice (<21), moderate usage of self-medication practice (21-35), and over usage of self-medication practice (>35). The reliability of the tool was obtained using the split-half method, and the score obtained was 0.97 which indicates that it is reliable.

Analysis

IBM SPSS Statistics for Windows, Version 20 (Released 2011; IBM Corp., Armonk, New York, United States) was used to analyze the data. The unpaired ‘t’ test was used to compare the level of knowledge of impact and practice of self-medication among adults in urban and rural communities. Karl Pearson correlation was used to find out correlation. The Chi-square test was used to find out the association.

## Results

Demographic variables 

Table 1 shows the frequency and percentage distribution of demographic variables among adults in urban and rural communities and the adults' details such as age, gender, religion, education, occupation, marital status, and monthly income.

**Table 1:** Socio-economic demographic variables among urban and rural communities (N=100)

Sl.no	Demographic variable	Urban adults		Rural adults	
		Frequency	Percentage	Frequency	Percentage
1.	Age in year 21-30 years	19	38.0	22	44.0
	31-40 years	15	30.0	15	30.0
	41-50 years	11	22.0	9	18.0
	51-60 years	5	10.0	4	8.0
2.	Gender Male	25	50.0	23	46.0
	Female	25	50.0	27	54.0
3.	Religion Hindu	28	56.0	38	76.0
	Muslim	10	20.0	6	12.0
	Christian	12	24.0	6	12.0
4.	Educational status No formal education	4	8.0	12	24.0
	Primary education	14	28.0	16	32.0
	Secondary education	15	30.0	16	32.0
	Higher secondary education	10	20.0	3	6.0
	Graduate and above post-graduated	7	14.0	3	6.0
5.	Occupational status Business	16	32.0	17	34.0
	Private employer	13	26.0	18	36.0
	Government employer	13	26.0	3	6.0
	Coolie	5	10.0	11	22.0
	Homemaker	3	6.0	1	2.0
6.	Marital status Single	15	30.0	13	26.0
	Married	35	70.0	37	74.0
7.	Monthly income <10,000	18	36.0	36	72.0
	10,000-15,000	12	24.0	6	12.0
	15001-20,000	9	18.0	6	12.0
	>20,000	11	22.0	2	4.0

Prevalence of self-medication

Table 2 shows the frequency and percentage distribution of prevalence of consuming self-medication among adults in urban and rural communities.

**Table 2:** Prevalence of self-medication among adults in urban and rural communities (N=100)

Sl.no	Prevalence	Urban adults				Rural adults			
		YES		NO		YES		NO	
		Freq	Percentage	Freq	Percentage	Freq	Percentage	Freq	percentage
1.	Are you consuming self-medication	50	100	-	-	50	100	-	-
2.	Reason for consuming self-medication Had a minor illness	40	80	10	20	34	68	16	32
	Lack of health facilities nearby area or locality	7	14	43	86	34	68	16	32
	Had an old prescription	38	76	12	24	8	16	42	84
3.	Sources of self-medication Family	40	80	10	20	28	56	22	44
	Pharmacy	38	76	12	24	32	64	18	36
	Friends	13	26	37	74	13	26	37	74
	Shops	8	16	42	84	38	76	12	24
4.	What are the minor ailments for consuming self-medication Headache	44	88	6	12	13	26	37	74
	Body pain	32	64	18	36	36	72	14	28
	Toothache	9	18	41	82	10	20	40	80
	Fever	37	74	13	26	33	66	17	34
	Common cold	41	82	9	18	30	60	20	40
	Other	19	38	31	62	12	24	38	78
5.	Most commonly used self-medicated drug Antibiotic	36	72	14	28	23	46	27	54
	Anti- allergic	26	52	24	48	12	24	38	76
	Painkiller	43	86	7	14	38	76	12	24
	Anti-diarrhoeal	30	60	20	40	11	22	39	78
	Anti-ulcer	38	76	12	24	37	74	13	26
	Eye drops	14	28	36	72	13	26	37	74
6.	How often you are taking self-medication Always	12		24		13		26	
	Sometimes	23		46		14		28	
	Every once in a while	8		16		6		12	
	Long duration	7		14		17		34	
7.	What a long duration your consuming self-medication Last two months	19		38		16		32	
	Last six months	3		6		17		34	
	Near a year	10		20		6		12	
	More than one year	18		36		11		22	

Comparison of knowledge and practice between urban and rural adults

Table 3 reveals that the unpaired t-test is 5.233. It was found to be statistically significant for knowledge of impact of self-medication at 0.05 level (i.e., p<0.05). Also, the unpaired test is 4.91; it was found to be statistically significant for practice regarding self-medication. It implied that knowledge of impact and practice of self-medication differ between adults in urban and rural communities at <0.05 level.

**Table 3:** Comparison of knowledge and practice between urban and rural communities (N=100)

S.no.	Variables	Max score	Urban adults		Rural adults		Unpaired t-test value	p-value
			Mean	SD	Mean	SD		
1.	Knowledge	20	9.46	2.27	6.74	2.43	5.233, S	p<0.05
2.	Practice	42	28.00	5.36	33.00	4.76	4.91, S	p<0.05

Level of knowledge of self-medication among adults in urban and rural communities

Twenty-seven urban adults (54%) and 44 rural adults (88%) had an inadequate level of knowledge of impact. Twenty (40%) urban adults and six (12%) rural adults had a moderate level of knowledge of impact. Three (6%) urban adults had an adequate level of knowledge of impact of self-medication, but in the rural community, none of the adults had adequate knowledge. This result shows that the majority of rural adults had inadequate knowledge of impact of self-medication.

Level of practice of self-medication among adults in urban and rural communities

Thirty-two urban adults (64%) and 18 rural adults (32%) had a moderate usage of self-medication practice. Fourteen urban adults (28%) and 32 rural adults (64%) had an over usage of self-medication practice. Four urban adults (8%) had a less usage of self-medication practice, but in the rural community, none of the adults had a less usage of self-medication practice.

Correlation between knowledge and practice among adults in urban and rural communities

There is a negative correlation, and it is found to be statistically significant between knowledge of impact and practice of self-medication among adults in urban (r =-0.924) and rural (r =-0.972) communities. Hence, the knowledge is decreased with the increase of practice of self-medication among both the populations.

## Discussion

The study was conducted to compare and assess the prevalence, knowledge of impact, and practice of self-medication among adults in urban and rural communities. In the current scenario, data show that in developing and developed countries most individuals practice self-medication. In India, the practice of self-medication is around 60-80% for treating illness [3]. Antibiotic consumption as a self-medication is common in the public to treat any kind of infection in the human body and has effects like drug resistance for the patients. Even educated and qualified personnel practice self-medication to manage minor illnesses. There should be proper guidelines for issuing medication in the pharmacy. The present study revealed that the majority of subjects in the urban adults, i.e. 27 (54%), and 44 (88%) in rural adults had an inadequate level of knowledge, and 20 urban adults (40%) and six rural adults (12%) had moderate and only minimal levels of knowledge, respectively. Regarding the level of practice, the majority of subjects in the urban adults had a maximum practice of self-medication (64%) and 18 (36%) in the rural adults had a moderate usage, and 14 urban adults (28%) and 32 rural adults (64%) had over usage of self-medication, respectively. From the comparison of urban and rural adults, knowledge independent 't' test value =5.23, and practice 't' value = 4.91, it can be seen that it is statistically significant at p<0.05. Regarding the correlation between knowledge and practice, there was negative correlation found and it was statistically significant at p<0.05.

The findings of a similar study were assessed to evaluate the pattern of self-medication practice among first-year and third-year medical students. Awareness about self-medication was statistically significantly higher in the third year than in the first-year medical students. Drugs most commonly practiced by self-medication were antipyretics and analgesics. The result showed that third-year students had better knowledge than first-year students regarding the practice of self-medication [5].

The current study found that the most common drug used was painkillers, followed by antibiotics and anti-ulcers. The main reason for consuming medication for subjects was a minor illness and a source for practicing self-medication through the family members in the urban and referred shops in the rural. The majority of the subjects consume self-medication due to headaches in the case of urban adults and body pain in the case of rural adults. 

Nurses are accessible and approachable professionals in the health care delivery system to convey the adverse effect of consuming self-medication among individuals and the community. In the present scenario, the practice of self-medication increases and its adverse reaction causes vital organ damage. This is the most common of all the age groups. Nurses can organize health education programs, awareness rallies, and mass awareness campaigns at various levels in the communities, hospitals, and colleges to minimize the consumption of self-medication.

The study has a few limitations. The sample size was 100, so the findings cannot be generalized. The study was conducted among urban and rural adults on knowledge and practice of self-medication. Their outcome variables may vary depending on their educational background and locality. 

## Conclusions

The study concluded that the majority of urban adults had better knowledge of self-medication as compared to rural adults. Regarding the practice of self-medication, higher percentages were found among rural adults. There should be proper guidelines to control the consumption of self-medication in the community. The adverse reaction rate to self-medication has increased gradually, and its negative impacts were identified at a higher percentage. The study recommends that there is a need to conduct many studies, like public awareness campaigns, education, and group discussions in this area to make the public aware. 
